# A Meta-Analysis of fMRI Activation Studies of Ketamine in Healthy Participants

**DOI:** 10.1192/j.eurpsy.2024.198

**Published:** 2024-08-27

**Authors:** J. H. Shepherd, A. Hickman, C. Baten, A. M. Klassen, G. Zamora, E. Johnson-Venegas, S. S. Madugula, E. Woo, J. A. Miller, M. D. Sacchet, D. W. Hedges, C. H. Miller

**Affiliations:** ^1^Department of Psychology, California State University, Fresno, Fresno; ^2^School of Medicine, Stanford University, Stanford; ^3^Department of Psychology, Palo Alto University, Palo Alto; ^4^Department of Psychiatry, Massachusetts General Hospital, Harvard Medical School, Boston; ^5^Department of Psychology, Brigham Young University, Provo, Provo, United States

## Abstract

**Introduction:**

There has been rapidly growing interest in understanding the pharmaceutical and clinical properties of psychedelic and dissociative drugs, with a particular focus on ketamine. This compound, long known for its anesthetic and dissociative properties, has garnered attention due to its potential to rapidly alleviate symptoms of depression, especially in individuals with treatment-resistant depression (TRD) or acute suicidal ideation or behavior. However, while ketamine’s psychopharmacological effects are increasingly well-documented, the specific patterns of its neural impact remain a subject of exploration and basic questions remain about its effects on functional activation in both clinical and healthy populations.

**Objectives:**

This meta-analysis seeks to contribute to the evolving landscape of neuroscience research on dissociative drugs such as ketamine by comprehensively examining the effects of acute ketamine administration on neural activation, as measured by functional magnetic resonance imaging (fMRI), in healthy participants.

**Methods:**

We conducted a meta-analysis of existing fMRI activation studies of ketamine using multilevel kernel density analysis (MKDA). Following a comprehensive PubMed search, we quantitatively synthesized all published primary fMRI whole-brain activation studies of the effects of ketamine in healthy subjects with no overlapping samples (N=18). This approach also incorporated ensemble thresholding (α=0.05-0.0001) to minimize cluster-size detection bias and Monte Carlo simulations to correct for multiple comparisons.

**Results:**

Our meta-analysis revealed statistically significant (p<0.05-0.0001; FWE-corrected) alterations in neural activation in multiple cortical and subcortical regions following the administration of ketamine to healthy participants (N=306).

**Conclusions:**

These results offer valuable insights into the functional neuroanatomical effects caused by acute ketamine administration. These findings may also inform development of therapeutic applications of ketamine for various psychiatric and neurological conditions. Future studies should investigate the neural effects of ketamine administration, including both short-term and long-term effects, in clinical populations and their relation to clinical and functional improvements.

**Disclosure of Interest:**

None Declared

